# Modeling the transmission dynamics of delayed pneumonia-like diseases with a sensitivity of parameters

**DOI:** 10.1186/s13662-021-03618-z

**Published:** 2021-10-20

**Authors:** Muhammad Naveed, Dumitru Baleanu, Ali Raza, Muhammad Rafiq, Atif Hassan Soori, Muhammad Mohsin

**Affiliations:** 1 Department of Mathematics, Air University, PAF Complex E-9, Islamabad, Pakistan; 2 Department of Mathematics, Cankaya University, 06530 Balgat, Ankara Turkey; 3 Institute of Space Sciences, Magurele-Bucharest, Romania; 4 Department of Medical Research, China Medical University Hospital, China Medical University, Taichung, Taiwan; 5 Department of Mathematics, Govt. Maulana Zafar Ali Khan Graduate College Wazirabad, Punjab Higher Education Department (PHED), Lahore, 54000 Pakistan; 6 Department of Mathematics, National College of Business Administration and Economics, Lahore, 54660 Pakistan; 7 Department of Mathematics, Faculty of Sciences, University of Central Punjab, Lahore, Pakistan; 8 Department of Mathematics, Technische Universitat Chemnitz, Chemnitz, Germany

**Keywords:** Pneumonia disease, Delayed model, Stability analysis, Numerical simulations

## Abstract

Pneumonia is a highly transmitted disease in children. According to the World Health Organization (WHO), the most affected regions include South Asia and sub-Saharan Africa. 15% deaths of children are due to pneumonia. In 2017, 0.88 million children were killed under the age of five years. An analysis of pneumonia disease is performed with the help of a delayed mathematical modelling technique. The epidemiological system contemplates subpopulations of susceptible, carriers, infected and recovered individuals, along with nonlinear interactions between the members of those subpopulations. The positivity and the boundedness of the ongoing problem for nonnegative initial data are thoroughly proved. The system possesses pneumonia-free and pneumonia existing equilibrium points, whose stability is studied rigorously. Moreover, the numerical simulations confirm the validity of these theoretical results.

## Literature survey

In 2014, Mochan et al. presented the interhost immune response to bacterial pneumonia infection in murine strains in a simple ordinary differential equation model dynamically [1]. Drusano et al., in 2014, investigated the effects of granulocytes to eradicate bacterial pathogens, and there was no role of antimicrobial therapy [2]. In 2015, Ndelwa et al. expressed the dynamic properties for the transfer of pneumonia along with screening and medication mathematically and analysed to know the transmission and effects [3]. Kosasih et al., in 2015, analysed a mathematical model of cough sounds by wavelet-based crackle detection work for rapid diagnosis of bacterial pneumonia in children [4]. In 2016, César et al. estimated particulate matter in a model mathematically and medications for both pneumonia and asthma in children among the population [5]. Marchello et al., in 2016, gave atypical bacterial pathogens as the main causes for lower respiratory diseases like cough, bronchitis, CAP and analysed them [6]. In 2017, Cheng et al. provided an IAV-SP model mathematically and dynamically. A quantitative risk-assessment framework to improve respiratory health due to COPD gave the hope for improvements [7]. Kosasih et al., in 2017, gave a simple mathematical model showing the analysis of measurements for pneumonia diagnosis among children clinically [8]. Tilahun et al., in 2017, proposed a deterministic nonlinear model mathematically and analysed optical control strategies for the bacterial disease pneumonia; results are shown graphically [9]. In 2018, Raj et al. analysed the classification of asthma and pneumonia based upon mathematical features of cough sound in the poor regions of the population [10]. Kizito et al., in 2018, presented a mathematical model that shows the control of pneumonia spread by bacteria. Also, the dynamics of treatment and formulation of vaccines were given [11]. In 2018, Mbabazi et al. investigated a mathematical model nonlinearly that described the modelling of within-host coinfection influenza A virus and pneumonia [12]. Tilahun et al., in 2018, proposed a coinfection model for pneumonia-typhoid and mathematically analysed their characteristic relationship in case of cure and medical strategies [13]. In 2019, Tilahun et al. described a model of pneumonia-meningitis coinfection with the help of ordinary differential equations and some of the theorems. It explained different techniques for disease clearance [14]. Diah et al., in 2019, reviewed mathematical models of pneumonia dynamically followed by research in the past. An alternative method was proposed to estimate the risk of pneumonia [15]. In 2019, Kosasih et al. explained the main cause of pneumonia affecting children in early childhood in poor regions of the world [8]. In 2019, Tilahun et al. analysed a coinfection mathematical model for pneumonia and bacterial meningitis [16]. Mbabazi et al., in 2019, proposed a pneumococcal pneumonia model with time delays mathematically, Hopf-bifurcation was analysed [17]. In 2020, Otoo et al. analysed a model of pneumonia spread by bacteria. The analysis determined the effects of vaccination to control the disease [18]. In 2020, Zephaniah et al. presented the dynamics of mathematical models for pneumonia, showing the result graphically [19]. Ming et al., in 2020, described the spread of coronavirus pneumonia in Wuhan, China, and discussed the increasing cases of infected people [20]. In 2020, Jung et al. demonstrated the observations using different clinical tests and showed the cause of disease, a novel pathogen [21]. Adams et al., in 2020, showed the progress regarding pneumonia prevention and different strategies to treat and overcome bacterial pneumonia [22]. In 2013, Ong’ala et al. developed a mathematical model for bacteremic pneumonia among children under five years. Using stability of equilibrium points and bifurcation, they analysed the reducing ways or the transfer rates between the carriers and the infected class [23]. Minuci et al. presented the review of mathematical modelling of the inflammatory response in lungs infections and injuries. They emphasised that mathematical modelling is a great tool for understanding infectious diseases [24]. Huttinger et al., in 2017, developed a mechanistic mathematical model explaining the dynamic relationship between streptococcus pneumonia (Sp), immune cells and epithelial tissues for the better understanding of complex dynamically changing host–Sp interaction. They claimed that their model provides help to plan better disease management strategies from the diagnostic and treatment perspective [25]. In 2021, Wafula et al. presented an article describing optimal control treatment by developing a deterministic mathematical model pneumonia–HIV coinfection incorporating the use of anti-pneumonia and ART treatment interventions as controls [26]. Oluwatobi et al. studied the effect of treatment of pneumonia infection and investigated the basic and effective reproduction numbers existence and the stability of equilibrium points [27]. Epidemiology has a significant role in different disciplines like medicine, engineering, chemistry, physics, economics and many more. A diseases model is investigated with the help of well-known branches of mathematics like spatio-temporal, stochastic, fractional and fractal fractional [28–48].

Pneumonia is a terrible strain in developing regions such as Asia, Africa and Europe. Pneumonia vaccination has been introduced in developed countries, and even the United States is the largest donor for nefarious purposes. Delay modelling is a more general form of deterministic modelling. The delayed analysis is a more realistic, close nature and authentic tool to control infectious diseases. For example, in the recent strain of coronavirus, the only control strategy is delaying tactics like public holidays, restriction in travel, facemask, hand sanitiser, social distancing etc. This article claims that precautionary measures or delay tactics are the best revenge to control pneumonia-like diseases other than vaccination or medication etc. That is a reason why we move in delay modelling.

The paper strategy is as follows: Sect. 1 presents literature survey regarding pneumonia-like diseases. Section 2 deals with the formulation of the delayed model and its mathematical analysis. In Sects. 3 and 4 the stability of the model is described locally and globally, respectively. Section 5 presents the sensitivity of the parameters involved in the model. In Sect. 6 the numerical simulations with their results are given. Section 7 contains the concluding remarks of the work.

## Formulation of the model

**Figure 1:**
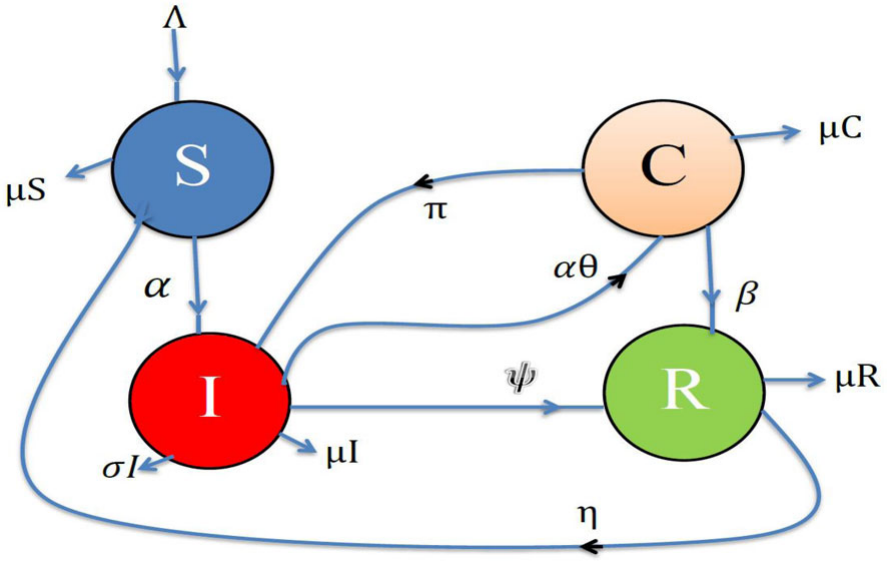
Flow chart of the pneumonia disease model

The formulation of the model is based on the theory of population dynamics. The population $N ( t )$ is the sum of the following classes: susceptible $S ( t )$, carriers $C ( t )$, infected $I ( t )$ and recovered $R ( t )$. The modelling of pneumonia disease based on the law of mass action is performed (see Fig. 1). Furthermore, some assumptions are fixed during the delay modelling as follows:

- Newly recruited individuals are assumed to be in the susceptible class.
- The birth and death rates are approximately equal.
- Vertical transmission is to be considered.
- Susceptible types make attraction with the infected and carriers classes at any time $( {t}-\tau )$.
- Natural delay is assumed.
- An artificial delay term like ${e}^{-\mu \tau }, \tau \geq 0$ (a decay term) is used for controlling the epidemic.
- Once recovered, they may have chances to get the disease again.
- The carriers may recover directly due to their internal immunity.
- The carriers and infected classes have inverse interactions with each other.
- Two types of death rates are considered: natural and due to the disease.

Table 1 presents the physical relevance of the constants as follows.

**Table 1:** Physical applicability of the model

Parameters	Descriptions	Values (per day)/Source [11]
*μ*	The natural mortality rate of individuals.	0.5 (Assumed)
Λ	Recruitment rate.	0.5 (Assumed)
*θ*	The proportion of sensitive people involved in the careers.	0.536
*σ*	Rate of mortality due to the disease.	0.53
*β*	The recovery rate of the carriers.	0.515
*ψ*	Rate of recovery of children who are infected with pneumonia.	0.614
*π*	Signifies the rate of developing symptoms by the carriers	0.7096
*η*	Rate of the treated individuals becoming susceptible.	0.00641
*ω*	Represented as the coefficient of transmission for the subgroup involving carriers.	0.1124
*δ*	The transmission rate of the disease among the population.	2 (PFE)
		2.5 (PEE)
*p*	The rate of contact is quite effective due to infection.	0.89–0.99
*κ*	Nonlinear contact rate.	≥1

Based on the assumptions, the continuous model is defined with the help of the law of mass action. The nonlinear delay differential equations (DDEs) present the transmission flow of pneumonia type diseases as follows:

1 $$\begin{aligned} &S ' ({t})=\Lambda - \kappa p {I} ( {t}- \tau ) {S} ( {t}-\tau ) {e}^{-\mu \tau } -\kappa p\omega {C} ( {t}-\tau ) {S} ( {t}-\tau ) {e}^{-\mu \tau } -\mu {S} ( {t} ) +\eta {R} ( {t} ), \end{aligned}$$

2 $$\begin{aligned} &C ' ({t})= \theta \kappa p {I} ( {t}-\tau ) {S} ( {t}-\tau ) {e}^{-\mu \tau } +\theta \kappa p\omega {C} ( {t}-\tau ) {S} ( {t}-\tau ) {e}^{-\mu \tau } - ( \mu +\beta +\pi ) {C} ( {t} ), \end{aligned}$$

3 $$\begin{aligned} &I ' ( {t} ) = \kappa p {I} ( {t}-\tau ) {S} ( {t}-\tau ) {e}^{-\mu \tau } + \kappa p\omega {C} ( {t}-\tau ) {S} ( {t}-\tau ) {e}^{-\mu \tau } \\ &\phantom{I ' ( {t} ) =}{} - \theta \kappa p {I} ( {t}-\tau ) {S} ( {t}-\tau ) {e}^{-\mu \tau } -\theta \kappa p\omega {C} ( {t}-\tau ) {S} ( {t}-\tau ) {e}^{-\mu \tau } +\pi {C} ( {t} ) \\ &\phantom{I ' ( {t} ) =}{}- ( \psi +\mu +\sigma ) {I} ( {t} ), \end{aligned}$$

4 $$\begin{aligned} &R ' ({t})= \beta C ( t ) + \psi {I} ( {t} ) - ( \mu +\eta ) {R} ( {t} ) \end{aligned}$$

with nonnegative initial conditions $S= S_{0}\geq0$, $C=C_{0}\geq0$, $I= I_{0}\geq0$, $R= R_{0}\geq0$ and ${t}\geq 0, \tau \leq t$.

### Model properties

To preserve the meaningful analysis of the model, all the variables $S ( t ), C ( t ), I ( t ), R ( t )$ must be nonnegative. Consequently, the outcomes are achieved after studying the model for any time $t \geq 0 \tau \leq t$ in a feasible region.

$$\begin{aligned} \mathcal{H}= \biggl\{ ( S, C, I, R ) \epsilon \mathbb{{R}}_{+}^{4}: N ( t ) \leq \frac{\Lambda }{\mu }, S \geq 0, {C} \geq 0, {I} \geq 0, {R} \geq 0 \biggr\} . \end{aligned}$$

#### Theorem 2.1

*The solutions* $({S}, {C}, {I}, {R}) \epsilon \mathbb{{R}}_{+}^{4}$ *of system* (1)*–*(4) *are positive at* ${t}\geq 0, \tau \leq {t}$ *with given nonnegative initial conditions*.

#### Proof

Obviously, system (1)–(4) is as follows:

$$\begin{aligned} & \frac{{dS}}{ {dt}} \bigg\vert _{{S}=0} = ( \Lambda -\eta {R} ) \geq 0, \qquad \frac{{dC}}{ {dt}} \bigg\vert _{{C}=0} = \theta \kappa p{IS} {e}^{-\mu \tau } \geq 0, \\ & \frac{{dI}}{ {dt}} \bigg\vert _{{I}=0} = \bigl[ \kappa p\omega {CS} ( 1-\theta ) {e}^{-\mu \tau } +\pi {C} \bigr] \geq 0, \qquad \frac{{dR}}{ {dt}} \bigg\vert _{{R}=0} = ( \beta C + \psi {I} ) \geq 0. \end{aligned}$$

Since all the parameters and derivatives of the model are nonnegative, system (1)–(4) admits a positive solution, as desired. □

#### Theorem 2.2

*The solutions* $({S}, {C}, {I}, {R}) \epsilon \mathbb{{R}}_{+}^{4}$ *of system* (1)*–*(4) *are bounded*.

#### Proof

Consider the population function:

$$\begin{aligned} &{N} ( {t} ) ={S} ( {t} ) +{C} ( {t} ) +{I} ( {t} ) +{S} ( {t} ), \\ &\frac{dN}{dt} =\Lambda -\mu ({S}+{C}+{I}+{R})- \sigma {I}, \qquad N=S+C+I+R, \\ &\frac{{dN}}{ {dt}} \leq \Lambda -\mu {N}. \end{aligned}$$

Using Gronwall’s inequality [49], we have

$$\begin{aligned} {N} ( {t} ) \leq {N} ( 0 ) {e}^{-\mu \tau } + \frac{\Lambda }{\mu },\quad {t}\geq 0, \end{aligned}$$

$\lim_{{it}\longrightarrow \infty } \operatorname{Sup} {N}({t})\leq \frac{\Lambda }{\mu }$, as desired. □

### Analysis of the model

This section shows a brief discussion of the equilibria of the pneumonia delayed model. We will discuss trivial pneumonia equilibrium ($\mathcal{P}_{n} TE - {D}_{0}$), pneumonia-free equilibrium ($\mathcal{P}_{n} FE - {D}_{1} $) and pneumonia existing equilibrium $( \mathcal{P}_{n} EE - {D}_{2} )$ given by

$$\begin{aligned} &{D}_{0} = \bigl( S^{0}, C^{0}, I^{0}, R^{0} \bigr) = ( 0,0,0,0 ),\qquad {D}_{1} = \bigl( S^{1}, C^{1}, I^{1}, R^{1} \bigr) = \biggl( \frac{\Lambda }{\mu },0,0,0 \biggr) \quad\text{and} \\ &{D}_{2} = \bigl( S^{*}, C^{*}, I^{*}, R^{*} \bigr), \\ &S^{*} = \frac{\Lambda }{\mu \mathcal{R}_{0}} \quad\text{or}\quad S^{*} = \frac{u_{1} u_{2}}{\kappa p[ u_{1} ( 1-\theta ) +\pi \theta +\omega \theta u_{2} ] {e}^{-\mu \tau }}, \\ &C^{*} = \frac{\Lambda \theta {u}_{2} ( \mu +\eta ) ( \mathcal{R}_{0} -1 )}{\mathcal{R}_{0} [ \{ u_{1} ( \mu +\eta ) -\eta \theta \beta \} {u}_{2} -\eta \psi \{ u_{1} ( 1-\theta ) +\pi \theta \} ]}, \\ &I^{*} = \frac{\Lambda ( \mu +\eta ) [ u_{1} ( 0.1-\theta ) +\pi \theta ] ( \mathcal{R}_{0} -1 )}{\mathcal{R}_{0} [ \{ u_{1} ( \mu +\eta ) -\eta \theta \beta \} {u}_{2} -\eta \psi \{ u_{1} ( 1-\theta ) +\pi \theta \} ]}, \\ &{R}^{*} = \frac{\Lambda [ \beta \theta {u}_{2} +\psi \{ u_{1} ( 1-\theta ) +\pi \theta \} ] ( \mathcal{R}_{0} -1 )}{\mathcal{R}_{0} [ \{ u_{1} ( \mu +\eta ) -\eta \theta \beta \} {u}_{2} -\eta \psi \{ u_{1} ( 1-\theta ) +\pi \theta \} ]}. \end{aligned}$$

### Reproduction number

The idea of reproduction number by using the next-generation matrix method is presented in [50]. The next-generation matrix method is implanted into system (1)–(4) to calculate the reproduction number $R_{0}$. After taking the carriers and infected classes from Eq. (1) to Eq. (4), along with the pneumonia-free equilibrium as follows:

$$\begin{aligned} \begin{bmatrix} C '\\ I ' \end{bmatrix} = \begin{bmatrix} \frac{\theta \kappa p\omega \Lambda {e}^{-\mu \tau }}{\mu } & \frac{\theta \kappa p\Lambda {e}^{-\mu \tau }}{\mu } \\ \frac{\kappa p\omega ( 1-\theta ) \Lambda {e}^{-\mu \tau }}{\mu } & \frac{\kappa p( 1-\theta ) \Lambda {e}^{-\mu \tau }}{\mu } \end{bmatrix} \begin{bmatrix} {C}\\ I \end{bmatrix} - \begin{bmatrix} u_{1} & 0\\ - \pi & u_{2} \end{bmatrix} \begin{bmatrix} {C}\\ I \end{bmatrix}, \end{aligned}$$

where $u_{1} = ( \mu +\beta +\pi )$ and $u_{2} = ( \psi +\mu +\sigma )$, $\theta _{1} = (1-\theta )$,

$$\begin{aligned} &A = \begin{bmatrix} \frac{\theta \kappa p\omega \Lambda {e}^{-\mu \tau }}{\mu } & \frac{\theta \kappa p\Lambda {e}^{-\mu \tau }}{\mu } \\ \frac{\kappa p\omega \theta _{1} \Lambda {e}^{-\mu \tau }}{\mu } & \frac{\kappa p\theta _{1} \Lambda {e}^{-\mu \tau }}{\mu } \end{bmatrix},\qquad B = \begin{bmatrix} u_{1} & 0\\ - \pi & u_{2} \end{bmatrix}, \\ &A B^{-1} \vert _{{D}_{1}} = \begin{bmatrix} \frac{\theta \kappa p\Lambda {e}^{-\mu \tau } ( \omega u_{2} +\pi )}{\mu u_{1} u_{2}} & \frac{\theta \kappa p\Lambda {e}^{-\mu \tau }}{\mu u_{2}}\\ \frac{\theta _{1} \kappa p\Lambda {e}^{-\mu \tau } ( \omega u_{2} +\pi )}{\mu u_{1} u_{2}} & \frac{\theta _{1} \kappa p\Lambda {e}^{-\mu \tau }}{\mu u_{2}} \end{bmatrix}. \end{aligned}$$

The spectral radius of $A B^{-1} \vert _{{D}_{1}}$ is called reproduction number and is defined as

$$\begin{aligned} \mathcal{R}_{0} = \frac{\kappa p\Lambda {e}^{-\mu \tau }}{{u}_{1} {u}_{2} \mu } \bigl[ \theta ( \omega {u}_{2} + \pi )+ \theta _{1} \bigr]. \end{aligned}$$

## Local stability

In this section, we examine the local stability of the model at the equilibrium of the model using the following recognised results.

The Jacobian matrix of system (1)–(4) and its elements are given below:

5 $$\begin{aligned} J_{\mathcal{P}_{n}} = \begin{bmatrix} J_{11} & J_{12} & J_{13} & J_{14}\\ J_{21} & J_{22} & J_{23} & J_{24}\\ J_{31} & J_{32} & J_{33} & J_{34}\\ J_{41} & J_{42} & J_{43} & J_{44} \end{bmatrix} \end{aligned}$$

$J_{11} = - \kappa p( {I}+\omega {C} ) {e}^{-\mu \tau } -\mu, J_{12} = -\kappa p\omega {S} {e}^{-\mu \tau }, J_{13} = - \kappa p {S} {e}^{-\mu \tau }, J_{14} = \eta , J_{21} =\theta \kappa p( {I}+{C} ) {e}^{-\mu \tau }, J_{22} =\theta \kappa p\omega {S} {e}^{-\mu \tau } - u_{1}, J_{23} =\theta \kappa p {S} {e}^{-\mu \tau }, J_{24} =0, J_{31} =\kappa p( {I}+\omega {C} ) \theta _{1} {e}^{-\mu \tau }, J_{32} =\kappa p\omega {S} \theta _{1} {e}^{-\mu \tau } +\pi, J_{33} =\kappa p {S} \theta _{1} {e}^{-\mu \tau } - u_{2}, J_{34} =0, J_{41} =0, J_{42} = \beta, J_{43} = \psi, J_{44} =- ( \mu +\eta )$.

### Theorem 3.1

*The pneumonia trivial equilibrium* ($\mathcal{P}_{n}$*TE*-$D_{0}$), $D_{0} = ( S^{0}, C^{0}, I^{0}, R^{0} ) =(0,0,0,0)$ *is locally asymptotically stable if* $\mathcal{R}_{0} =1$.

### Proof

The Jacobian matrix (5) at ${D}_{0} = ( {S}^{0}, {C}^{0}, {I}^{0}, {R}^{0} ) =(0,0,0,0)$ is as follows:

$$\begin{aligned} J_{\mathcal{P}_{n}} \vert _{{D}_{0}} = \begin{bmatrix} -\mu & 0 & 0 & \eta \\ 0 & - u_{1} & 0 & 0\\ 0 & \pi & - u_{2} & 0\\ 0 & \beta & \psi & - ( \mu +\eta ) \end{bmatrix}. \end{aligned}$$

The detailed proof is given in Appendix 1. So, by the Routh–Hurwitz criterion, the pneumonia trivial equilibrium point ($\mathcal{P}_{n} {TE}- {D}_{0}$) is locally asymptotically stable. □

### Theorem 3.2

*The pneumonia*-*free equilibrium*, $D_{1} = ( S^{1}, C^{1}, I^{1}, R^{1} ) = ( \frac{\Lambda }{\mu },0,0,0 )$ *is locally asymptotically stable if* $\mathcal{R}_{0} <1$.

### Proof

The Jacobian matrix (5) at $D_{1} = ( S^{1}, C^{1}, I^{1}, R^{1} ) = ( \frac{\Lambda }{\mu },0,0,0 )$ is as follows:

$$\begin{aligned} J_{\mathcal{P}_{n}} \vert _{{D}_{1}} = \begin{bmatrix} -\mu & \frac{-\kappa p\omega \Lambda {e}^{-\mu \tau }}{\mu } & \frac{- \kappa p\Lambda {e}^{-\mu \tau }}{\mu } & \eta \\ 0 & \frac{\theta \kappa p\omega \Lambda {e}^{-\mu \tau }}{\mu } - u_{1} & \frac{\theta \kappa p\Lambda {e}^{-\mu \tau }}{\mu } & 0\\ 0 & \frac{\kappa p\omega \Lambda \theta _{1} {e}^{-\mu \tau }}{\mu } +\pi & \frac{\kappa p\Lambda \theta _{1} {e}^{-\mu \tau }}{\mu } - u_{2} & 0\\ 0 & \beta & \psi & - ( \mu +\eta ) \end{bmatrix}. \end{aligned}$$

The detailed proof is given in Appendix 2 since all the eigenvalues are negative. Therefore, by Routh–Hurwitz criterion for the 2nd-degree polynomial, both fixed values of $x_{1}, x_{0} >0$ if $\mathcal{R}_{0} <1$. Hence the pneumonia-free equilibrium ($\mathcal{P}_{n} {FE}- {D}_{1}$) of system (1)–(4) is locally asymptotically stable. In other circumstances, if $\mathcal{R}_{0} >1$, then the Routh–Hurwitz condition does not hold. Thus, $D_{1}$ is unstable. □

### Theorem 3.3

*Pneumonia existing equilibrium* ($\mathcal{P}_{n} {EE}- {D}_{2}$), $D_{2} = ( S^{*}, C^{*}, I^{*}, R^{*} )$ *is locally asymptotically stable if* $\mathcal{R}_{0} >1$.

### Proof

The Jacobian matrix (5) at $D_{2} = ( S^{*}, C^{*}, I^{*}, R^{*} )$ is as follows:

$$\begin{aligned} J_{\mathcal{P}_{n}} \vert _{{D}_{2}} = \begin{bmatrix} - \kappa p( {I}^{*} +\omega {C}^{*} ) {e}^{-\mu \tau } -\mu & -\kappa p\omega {S}^{*} {e}^{-\mu \tau } & - \kappa p{S}^{*} {e}^{-\mu \tau } & \eta \\ \theta \kappa p( {I}^{*} + {C}^{*} ) {e}^{-\mu \tau } & \theta \kappa p\omega {S}^{*} {e}^{-\mu \tau } - u_{1} & \theta \kappa p{S}^{*} {e}^{-\mu \tau } & 0\\ \kappa p( {I}^{*} +\omega {C}^{*} ) \theta _{1} {e}^{-\mu \tau } & \kappa p\omega {S}^{*} \theta _{1} {e}^{-\mu \tau } +\pi & \kappa p{S}^{*} \theta _{1} {e}^{-\mu \tau } - u_{2} & 0\\ 0 & \beta & \psi & - ( \mu +\eta ) \end{bmatrix}. \end{aligned}$$

The detailed proof is given in Appendix 3. By the Routh–Hurwitz criterion for the 4th-degree polynomial, the given constraint has been verified if $\mathcal{R}_{0} >1$. Therefore pneumonia existing equilibrium ($\mathcal{P}_{n} {EE}- {D}_{2}$) of system (1)–(4) is locally asymptotically stable. □

## Global stability

The following theorems are presented for the system’s stability (1)–(4) in the global sense.

### Theorem 4.1

*The pneumonia trivial equilibrium* ($\mathcal{P}_{n} TE - D_{0}$), $D_{0} = ( S^{0}, C^{0}, I^{0}, R^{0} ) =(0,0,0,0)$ *is globally asymptotically stable if* $\mathcal{R}_{0} =1$.

### Proof

The Lyapunov function $\Omega: \mathcal{H}\rightarrow \mathbb{{R}}$ is defined as follows:

$$\begin{aligned} &\Omega = S + C + I + R,\quad \forall ( S, C, I, R ) \epsilon \mathcal{H}, \\ &\frac{d \Omega }{dt} = \frac{dS}{dt} + \frac{dC}{dt} + \frac{dI}{dt} + \frac{dR}{dt}, \\ &\frac{d \Omega }{dt} =\Lambda - \kappa p {IS} {e}^{-\mu \tau } -\kappa p\omega {CS} {e}^{-\mu \tau } -\mu {S}+\eta {R}+ \theta \kappa p {IS} {e}^{-\mu \tau } +\theta \kappa p\omega {CS} {e}^{-\mu \tau } \\ &\phantom{\frac{d \Omega }{dt} =}{}- ( \mu +\beta +\pi ) {C}+ \kappa p {IS} {e}^{-\mu \tau } + \kappa p\omega {CS} {e}^{-\mu \tau } - \theta \kappa p {IS} {e}^{-\mu \tau } -\theta \kappa p\omega {CS} {e}^{-\mu \tau } +\pi {C}\\ &\phantom{\frac{d \Omega }{dt} =}{}- ( \psi +\mu +\sigma ) {I}+ \beta C + \psi {I}- ( \mu +\eta ) {R}, \\ &\frac{d \Omega }{dt} =\Lambda -\mu N - \sigma I,\quad N=S+C+I+R, \\ &\frac{d \Omega }{dt} \leq \Lambda -\mu N,\quad N \leq \frac{\Lambda }{\mu },\\ &\frac{d \Omega }{dt} \leq 0, \quad \text{if }\mathcal{R}_{0} =1\text{ and }\frac{d \Omega }{dt} =0. \end{aligned}$$

Hence $D_{0}$ is globally asymptotically stable. □

### Theorem 4.2

*The pneumonia*-*free equilibrium* ($\mathcal{P}_{n}$*FE*-$D_{1}$), $D_{1} = ( S^{1}, C^{1}, I^{1}, R^{1} ) = ( \frac{\Lambda }{\mu },0,0,0)$ *is globally asymptotically stable if* $\mathcal{R}_{0} <1$.

### Proof

Define the Lyapunov function $U:\mathcal{H}\rightarrow \mathbb{{R}}$ as follows:

$$\begin{aligned} &U = \biggl( S - S^{1} - S^{1} \log \frac{S}{S^{1}} \biggr) + C + I + R,\quad\forall ( S, C, I, R ) \epsilon \mathcal{H}, \\ &\frac{dU}{dt} = \biggl( \frac{S - S^{1}}{S} \biggr) \frac{dS}{dt} + \frac{dC}{dt} + \frac{dI}{dt} + \frac{dR}{dt}, \\ &\frac{dU}{dt} = \biggl( \frac{S - S^{1}}{S} \biggr) \bigl[ \Lambda - \kappa p {IS} {e}^{-\mu \tau } -\kappa p\omega {CS} {e}^{-\mu \tau } -\mu {S}+\eta {R} \bigr] \\ &\phantom{\frac{d \Omega }{dt} =}{}+ \bigl[ \theta \kappa p {IS} {e}^{-\mu \tau } + \theta \kappa p\omega {CS} {e}^{-\mu \tau } - u_{1} {C} \bigr]\\ &\phantom{\frac{d \Omega }{dt} =}{} + \bigl[ \kappa p {IS} \theta _{1} {e}^{-\mu \tau } + \kappa p\omega {CS} \theta _{1} {e}^{-\mu \tau } +\pi {C}- u_{2} {I} \bigr] \\ &\phantom{\frac{d \Omega }{dt} =}{}+ \bigl[ \beta C + \psi {I}- ( \mu +\eta ) {R} \bigr]. \end{aligned}$$

The detailed proof is given in Appendix 4. $\frac{dU}{dt} \leq 0$ if $\mathcal{R}_{0} <1$ and $\frac{dU}{dt} =0$ if $S = S^{1}, C =0, I =0 \text{ and } R =0$. Since by the Lassalle invariance principle $D_{1}$ is the only unique trajectory of system (1)–(4), $D_{1}$ is globally asymptotically stable. □

### Theorem 4.3

*The pneumonia existence equilibrium* ($\mathcal{P}_{n}$*EE*-$D_{2}$), $D_{2} = ( S^{*}, C^{*}, I^{*}, R^{*} )$ *is globally asymptotically stable if* $\mathcal{R}_{0} >1$.

### Proof

Define the Volterra–Lyapunov function V $:\mathcal{H}\rightarrow \mathbb{{R}}$ as follows:

$$\begin{aligned} &V = \biggl( S - S^{*} - S^{*} \log \frac{S}{S^{*}} \biggr) + \biggl( C - C^{*} - C^{*} \log \frac{C}{C^{*}} \biggr) + \biggl( I - I^{*} - I^{*} \log \frac{I}{I^{*}} \biggr) \\ &\phantom{V =}{}+ \biggl( R - R^{*} - R^{*} \log \frac{R}{R^{*}} \biggr),\quad\forall ( S, C, I, R ) \epsilon \mathcal{H}. \\ &\frac{dV}{dt} = \biggl( 1- \frac{S^{*}}{S} \biggr) \frac{dS}{dt} + \biggl( 1- \frac{C^{*}}{C} \biggr) \frac{dC}{dt} + \biggl( 1- \frac{I^{*}}{I} \biggr) \frac{dI}{dt} + \biggl( 1- \frac{R^{*}}{R} \biggr) \frac{dR}{dt}. \end{aligned}$$

The detailed proof is given in Appendix 5. $\frac{dV}{dt} \leq 0$ if $\mathcal{R}_{0} >1$ and $\frac{dV}{dt} =0$ if $S = S^{*},{C}= C^{*}, {I}= I^{*} \text{and} R = R^{*}$. Since by the Lassalle invariance principle $D_{2}$ is the unique trajectory of system (1)–(4), $D_{2}$ is globally asymptotically stable. □

## Sensitivity analysis

We use the derivative-based local methods for the sensitivity analysis to take the partial derivatives of outputs concerning inputs as presented in [51]. The study highlights the importance of transmission rates that can change dynamics from pneumonia-free to pneumonia existing.

$$\begin{aligned} &\mathcal{P}_{n_{\kappa }} = \frac{\frac{\partial \mathcal{R}_{0}}{\mathcal{R}_{0}}}{\frac{\partial \kappa }{\kappa }} = \frac{\kappa }{\mathcal{R}_{0}} \times \frac{\partial \mathcal{R}_{0}}{\partial \kappa } =1>0, \qquad\mathcal{P}_{n_{p}} = \frac{\frac{\partial \mathcal{R}_{0}}{\mathcal{R}_{0}}}{\frac{\partial p}{ p}} = \frac{p}{\mathcal{R}_{0}} \times \frac{\partial \mathcal{R}_{0}}{\partial p} =1>0,\\ & \mathcal{P}_{n_{\Lambda }} = \frac{\frac{\partial \mathcal{R}_{0}}{\mathcal{R}_{0}}}{\frac{\partial \Lambda }{\Lambda }} = \frac{\Lambda }{\mathcal{R}_{0}} \times \frac{\partial \mathcal{R}_{0}}{\partial \Lambda } =1>0, \\ &\mathcal{P}_{n_{\theta }} = \frac{\frac{\partial \mathcal{R}_{0}}{\mathcal{R}_{0}}}{\frac{\partial \theta }{\theta }} = \frac{\theta }{\mathcal{R}_{0}} \times \frac{\partial \mathcal{R}_{0}}{\partial \theta } = \frac{\theta }{\mathcal{R}_{0}} \times \kappa p\Lambda {e}^{-\mu \tau } \biggl[ \frac{ ( \omega {u}_{2} + \pi ) - {u}_{1}}{{u}_{1} {u}_{2} \mu } \biggr],\\ & \mathcal{P}_{n_{\omega }} = \frac{\frac{\partial \mathcal{R}_{0}}{\mathcal{R}_{0}}}{\frac{\partial \omega }{\omega }} = \frac{\omega }{\mathcal{R}_{0}} \times \frac{\partial \mathcal{R}_{0}}{\partial \omega } = \frac{\omega }{\mathcal{R}_{0}} \times \biggl[ \frac{\theta \kappa p\Lambda {e}^{-\mu \tau }}{{u}_{1} \mu } \biggr] >0, \\ &\mathcal{P}_{n_{\tau }} = \frac{\frac{\partial \mathcal{R}_{0}}{\mathcal{R}_{0}}}{\frac{\partial \psi }{\psi }} = \frac{\psi }{\mathcal{R}_{0}} \times \frac{\partial \mathcal{R}_{0}}{\partial \psi } = \frac{\psi }{\mathcal{R}_{0}} \times \frac{\kappa p\Lambda {e}^{-\mu \tau } \theta \omega }{{u}_{1} {u}_{2} \mu } \biggl[ 1- \frac{ [ \theta ( \omega {u}_{2} + \pi ) + {u}_{1} ( 1- \theta ) ]}{\theta \omega {u}_{2}} \biggr] >0, \\ &\mathcal{P}_{n_{\mu }} = \frac{\frac{\partial \mathcal{R}_{0}}{\mathcal{R}_{0}}}{\frac{\partial \mu }{\mu }} = \frac{\mu }{\mathcal{R}_{0}} \times \frac{\partial \mathcal{R}_{0}}{\partial \mu }\\ &\phantom{\mathcal{P}_{n_{\mu }} } =- \frac{\mu }{\mathcal{R}_{0}} \times \kappa p\Lambda {e}^{-\mu \tau } \biggl[ \frac{\tau [ \theta ( \omega {u}_{2} + \pi ) + {u}_{1} ( 1- \theta ) - \theta \omega - ( 1- \theta ) ]}{{u}_{1} {u}_{2} \mu }\\ & \phantom{\mathcal{P}_{n_{\mu }} =}{}+ \frac{ [ \theta ( \omega {u}_{2} + \pi ) + {u}_{1} ( 1- \theta ) ] [ {u}_{1} {u}_{2} +\mu {u}_{1+} \mu {u}_{2} ]}{ ( {u}_{1} {u}_{2} \mu )^{2}} \biggr] < 0, \\ &\mathcal{P}_{n_{\sigma }} = \frac{\frac{\partial \mathcal{R}_{0}}{\mathcal{R}_{0}}}{\frac{\partial \sigma }{\sigma }} = \frac{\sigma }{\mathcal{R}_{0}} \times \frac{\partial \mathcal{R}_{0}}{\partial \sigma } = \frac{\sigma }{\mathcal{R}_{0}} \times \frac{\kappa p\Lambda {e}^{-\mu \tau } \theta \omega }{{u}_{1} {u}_{2} \mu } \biggl[ 1- \frac{ [ \theta ( \omega {u}_{2} + \pi ) + {u}_{1} ( 1- \theta ) ]}{\theta \omega {u}_{2}} \biggr] >0, \\ &\mathcal{P}_{n_{\pi }} = \frac{\frac{\partial \mathcal{R}_{0}}{\mathcal{R}_{0}}}{\frac{\partial \pi }{\pi }} = \frac{\pi }{\mathcal{R}_{0}} \times \frac{\partial \mathcal{R}_{0}}{\partial \pi } = \frac{\pi }{\mathcal{R}_{0}} \times \frac{\kappa p\Lambda {e}^{-\mu \tau } \theta \omega }{{u}_{1} {u}_{2} \mu } \biggl[ 1- \frac{ [ \theta ( \omega {u}_{2} + \pi ) + {u}_{1} ( 1- \theta ) ]}{{u}_{1}} \biggr] >0, \\ &\mathcal{P}_{n_{\beta }} = \frac{\frac{\partial \mathcal{R}_{0}}{\mathcal{R}_{0}}}{\frac{\partial \beta }{\beta }} = \frac{\beta }{\mathcal{R}_{0}} \times \frac{\partial \mathcal{R}_{0}}{\partial \beta } = \frac{\beta }{\mathcal{R}_{0}} \times \frac{\kappa p\Lambda {e}^{-\mu \tau } \theta \omega }{{u}_{1} {u}_{2} \mu } \biggl[ 1- \frac{ [ \theta ( \omega {u}_{2} + \pi ) + {u}_{1} ( 1- \theta ) ]}{{u}_{1} ( 1- \theta )} \biggr] >0. \end{aligned}$$

After that, the conclusion from the results mentioned above is that $\kappa, p,\Lambda, \theta, \omega, \psi,\sigma,\pi,\beta $ are sensitive and *μ* is not sensitive, as desired.

## Numerical simulations and results

Numerical simulations are presented by using the command build code of MATLAB software like DDE’s-23. The parameters have been taken from the scientific literature, as shown in [11] (see Table 1).

### Example 1

(Results on pneumonia-free equilibrium ($\mathcal{P}_{n}$EE-$D_{1}$) without delay)

**Figure 2:**
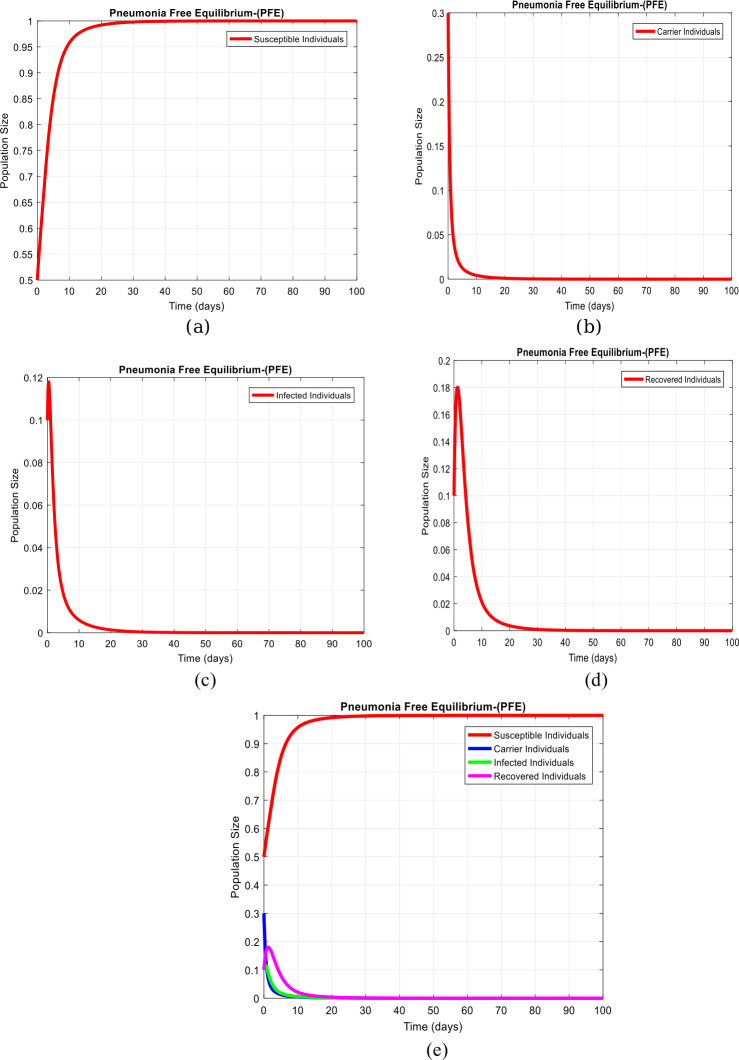
Subpopulations plots concerning the time of system (1)–(4) at the pneumonia-free equilibrium of the model when $\tau =0$

Figure 2(a) to (d) shows the solution at the pneumonia-free equilibrium ($\mathcal{P}_{n}$EE-$D_{1} $), when $\tau =0$, $D_{1} = ( S^{1}, C^{1}, I^{1}, R^{1} ) = ( 1,0,0,0 )$ with the nonnegative initial data and parameters presented in Table 1. Therefore, the value of the reproduction number is $R_{0}< 1$. Moreover, Fig. 2(e) shows the combined behaviour of the system.

### Example 2

(Simulation on pneumonia existing equilibrium ($\mathcal{P}_{n}$EE-$D_{2}$) without delay)

**Figure 3:**
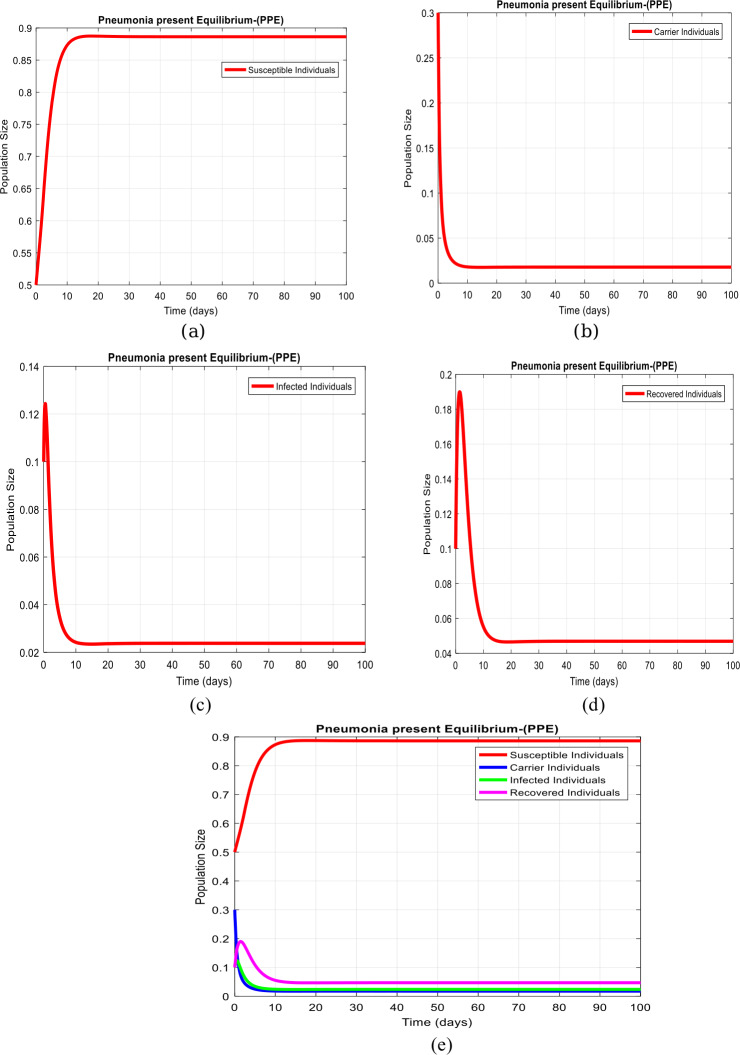
Time plots at the pneumonia existing equilibrium when $\tau =0$

Fig. 3(a) to (d) displays the solution of system (1)–(4) at the pneumonia existing equilibrium ($\mathcal{P}_{n}$EE-$D_{2} $), when $\tau =0$, $D_{2} = ( S^{*}, C^{*}, I^{*}, R^{*} ) = ( 0.8863,0.01776,0.0238,0.04692 )$. Therefore, the value of the reproduction number is $R_{0} >1$. Moreover, Fig. 3(e) shows the combined behaviour of the system.

### Example 3

(Results on pneumonia existing equilibrium)

**Figure 4:**
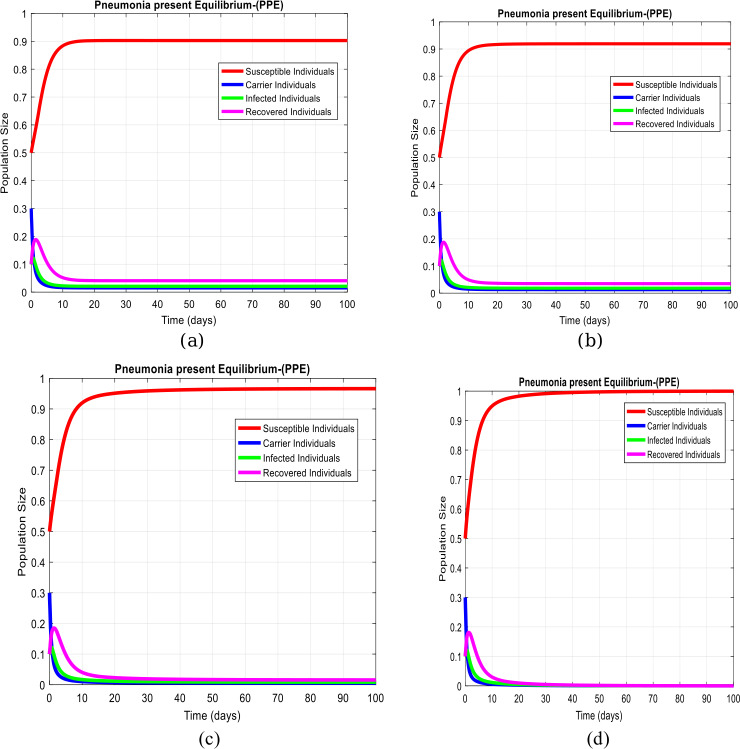
Subpopulations plots concerning the time of the system at the pneumonia existing equilibrium with practical uses of different values of delay tactics like $\tau =0.1,0.2,0.5,1$ respectively

In this section, we have taken the effect of system (1)–(4) with active practices of artificial delay tactics. Anti-pneumonia ability could be increased as observed in Fig. 4(a) to (d). On the other hand, we can keep the infectivity of pneumonia patients decreasing and even moving to zero.

### Example 4

(Behavior of reproduction number with the efficiency of delay tactics)

**Figure 5:**
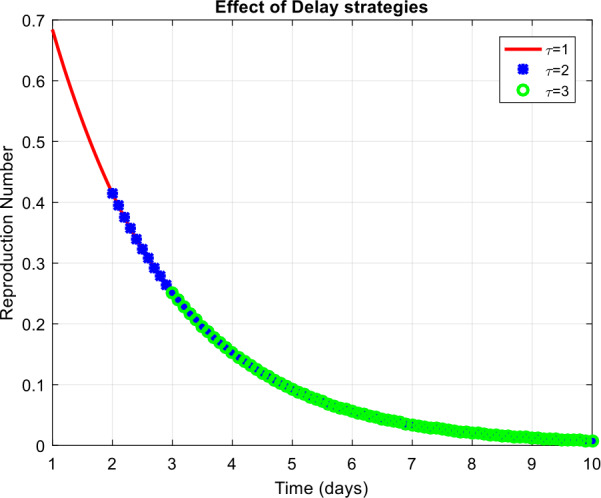
Simulation of the reproduction number with different values of the delay parameter

In Fig. 5, let $\tau =1,2,3$. As apparent, the value decreases, which changes the dynamics of the system of pneumonia disease from prevailing scenario to disease-free equilibrium.

### Example 5

(Behavior of infective class at different values of the delay parameter)

**Figure 6:**
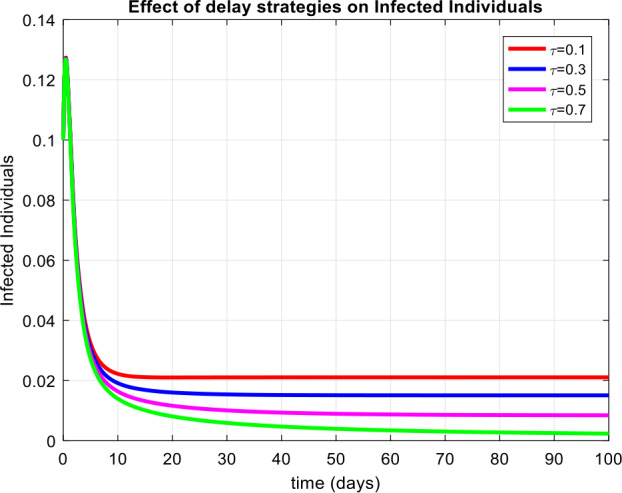
Subpopulation of the infected class concerning time with different delay values

Fig. 6 exhibits that with the increase in the value of the delayed parameter, the infectivity reduces gradually and is controlled.

### Example 6

(D-simulations of the system)

**Figure 7:**
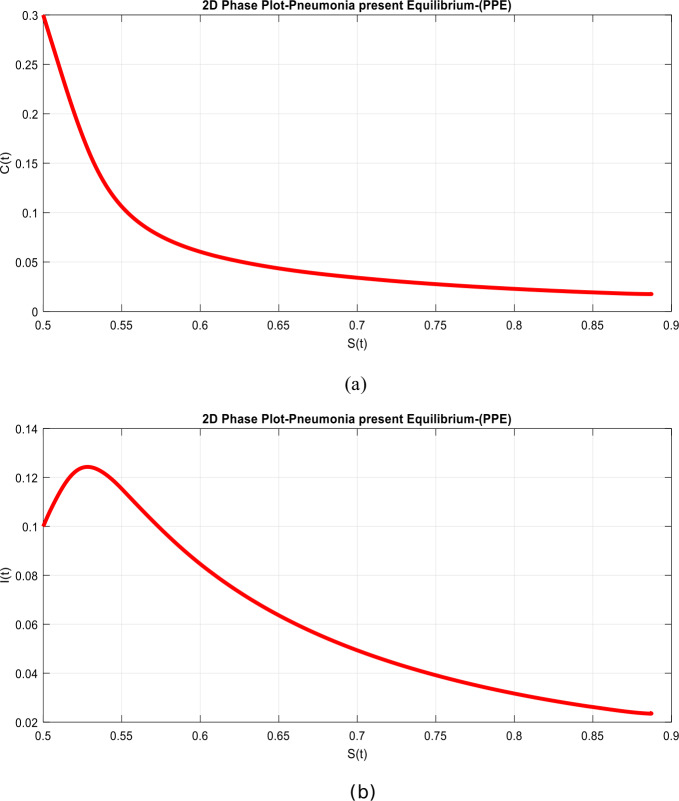
Time plots of the system in two-dimensional way when $\tau =0$

**Figure 8:**
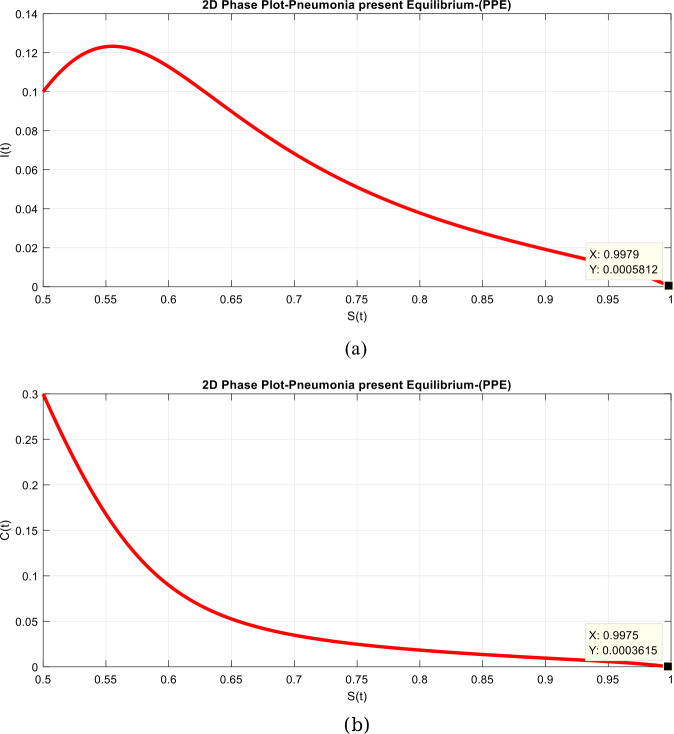
Time plots of the system in two-dimensional way when $\tau =1$

2D graphics are widely used in animation, providing a realistic but flat view of movement on the screen. We plotted the two-dimensional performance of the model with different subclasses for a better interpretation of the dynamics of pneumonia disease, as presented in Fig. 7(a)–(b) and 8(a)–(b) with and without delay effect, respectively, as desired.

## Conclusion

In this article, we investigated the mathematical analysis of pneumonia delayed epidemic models with reliable delay strategies. The model is based on four types of subpopulations like the susceptible, the carriers, the infected and the recovered. Dynamical analysis of the model includes positivity, boundedness, equilibria and threshold parameter. The sensitivity of the parameters is one of the outcomes of the model. Linearisation of the model is developed by well-known results like the Jacobian and Routh–Hurwitz criterion. Lyapunov theory and the Lassale invariance principle are investigated for the global stability of the model. The following artificial control measures could support the eradication of the disease in the population: the cleanness of hands, extensive use of fruits and vegetables, calm sleeping, staying away from smoking, vaccination to the children under five years and being aware of your general health.

## Data Availability

All data files are available.

## Appendix 1

Consider $\vert J_{\mathcal{P}_{n}} \vert _{{D}_{0}} - \lambda I \vert =0$

$$|J_{P_{n}}{|}_{D_{0}}-\lambda I|=\left|\begin{array}{cccc} -\mu -\lambda & 0 & 0 & \eta \\ 0 & -u_{1}-\lambda & 0 & 0\\ 0 & \pi & -u_{2}-\lambda & 0\\ 0 & \beta & \psi & -(\mu +\eta )-\lambda \end{array}\right|=0.$$

By solving the above determinant, we have the negative eigenvalues

$$\begin{aligned} \lambda _{1} =-\mu < 0,\qquad \lambda _{2} =- u_{1} < 0,\qquad \lambda _{3} =- u_{2} < 0, \qquad\lambda _{4} =- ( \mu + \eta ) < 0. \end{aligned}$$

## Appendix 2

Consider $\vert J_{\mathcal{P}_{n}} \vert _{{D}_{1}} - \lambda I \vert =0$

$$|J_{P_{n}}{|}_{D_{1}}-\lambda I|=\left|\begin{array}{cccc} -\mu -\lambda & \frac{-\kappa p\omega \Lambda e^{-\mu \tau }}{\mu } & \frac{-\kappa p\Lambda e^{-\mu \tau }}{\mu } & \eta \\ 0 & \frac{\theta \kappa p\omega \Lambda e^{-\mu \tau }}{\mu }-u_{1}-\lambda & \frac{\theta \kappa p\Lambda e^{-\mu \tau }}{\mu } & 0\\ 0 & \frac{\kappa p\omega \Lambda \theta_{1}e^{-\mu \tau }}{\mu }+\pi & \frac{\kappa p\Lambda \theta_{1}e^{-\mu \tau }}{\mu }-u_{2}-\lambda & 0\\ 0 & \beta & \psi & -(\mu +\eta )-\lambda \end{array}\right|=0.$$

The eigenvalues at ${D}_{1}$ are as follows:

$$\begin{aligned} \lambda _{1} =-\mu < 0,\qquad \lambda _{2} =- ( \mu +\eta ) < 0. \end{aligned}$$

And the polynomial $\lambda ^{2} + x_{1} \lambda + x_{0} =0$, where $x_{1} = ( u_{1} + u_{2} ) [ 1- \frac{\theta \kappa p\omega \Lambda {e}^{-\mu \tau } + \kappa p\Lambda \theta _{1} {e}^{-\mu \tau }}{\mu ( u_{1} + u_{2} )} ]$, $x_{0} = u_{1} u_{2} [ 1- \frac{\kappa p\Lambda \theta _{1} {e}^{-\mu \tau }}{\mu u_{2}} - \frac{\kappa p\Lambda {e}^{-\mu \tau }}{\mu } ( \frac{\omega u_{2} + \pi }{u_{1} u_{2}} ) ]$.

## Appendix 3

Consider $\vert J_{\mathcal{P}_{n}} \vert _{{D}_{2}} - \lambda I \vert =0$

$$\begin{aligned} \vert J_{\mathcal{P}_{n}} \vert _{{D}_{2}} - \lambda I \vert = \begin{bmatrix} - g_{1} -\mu -\lambda & - g_{2} & - g_{3} & \eta \\ g_{4} & g_{5} - u_{1} - \lambda & g_{6} & 0\\ g_{7} & g_{8} +\pi & g_{9} - u_{2} - \lambda & 0\\ 0 & \beta & \psi & - ( \mu +\eta ) -\lambda \end{bmatrix} =0, \end{aligned}$$

where $g_{1} = \kappa p( {I}^{*} +\omega {C}^{*} ) {e}^{-\mu \tau } $, $g_{2} =\kappa p\omega {S}^{*} {e}^{-\mu \tau } $, $g_{3} = \kappa p{S}^{*} {e}^{-\mu \tau } $, $g_{4} = \theta \kappa p( {I}^{*} + {C}^{*} ) {e}^{-\mu \tau } $, $g_{5} =\theta \kappa p\omega {S}^{*} {e}^{-\mu \tau } $, $g_{6} = \theta \kappa p{S}^{*} {e}^{-\mu \tau } $, $g_{7} = \kappa p( {I}^{*} +\omega {C}^{*} ) \theta _{1} {e}^{-\mu \tau } $, $g_{8} =\kappa p\omega {S}^{*} \theta _{1} {e}^{-\mu \tau } $, $g_{9} = \kappa p{S}^{*} \theta _{1} {e}^{-\mu \tau } $.

After solving $\vert J_{\mathcal{P}_{n}} \vert _{{D}_{2}} - \lambda I \vert =0 $, we have

$$\begin{aligned} \lambda ^{4} + y_{3} \lambda ^{3} + y_{2} \lambda ^{2} + y_{1} \lambda + y_{0} =0, \end{aligned}$$

where $y_{3} = [ ( \mu +\eta ) - g_{5} + u_{1} - g_{9} + u_{2} + g_{1} + \mu ]$,

$$\begin{aligned} &y_{2} = \bigl[ ( g_{5} - u_{1} ) ( g_{9} - u_{2} ) + g_{2} g_{4} + g_{3} g_{7} - ( \mu +\eta ) ( g_{5} - u_{1} + g_{9} - u_{2} - g_{1} - \mu )\\ &\phantom{y_{2} =}{} - ( g_{1} + \mu ) ( g_{1} - u_{1} + g_{9} - u_{2} ) - g_{6} ( g_{8} +\pi ) \bigr], \\ &y_{1} = \bigl[ ( g_{1} + \mu ) ( g_{5} - u_{1} ) ( g_{9} - u_{2} ) + g_{2} g_{4} ( g_{5} - u_{2} ) + g_{2} g_{6} g_{7} + g_{3} g_{4} ( g_{8} +\pi ) \\ &\phantom{y_{1} =}{}+ g_{3} g_{7} ( g_{5} - u_{1} ) - ( \beta g_{4} + \psi g_{7} ) - ( \mu + \eta ) \bigl\{ ( g_{1} +\mu ) ( g_{5} - u_{1} + g_{9} - u_{2} ) \\ &\phantom{y_{1} =}{}- ( g_{5} - u_{1} ) ( g_{9} - u_{2} ) + g_{6} ( g_{8} +\pi ) - g_{2} g_{4} - g_{3} g_{7} \bigr\} - g_{6} ( g_{8} +\pi ) ( g_{1} + \mu ) \bigr], \\ &y_{0} = \bigl[ \eta \bigl\{ \beta g_{4} ( g_{9} - u_{2} ) + \psi g_{7} ( g_{5} - u_{2} ) + \beta g_{6} g_{7} - \psi g_{4} ( g_{8} +\pi ) \bigr\} \\ &\phantom{y_{0} =}+ ( \mu +\eta ) \bigl\{ ( g_{1} +\mu ) ( g_{5} - u_{1} ) ( g_{9} - u_{2} ) - g_{6} ( g_{8} +\pi ) ( g_{1} + \mu ) \\ &\phantom{y_{0} =}{}+ g_{2} g_{4} ( g_{5} - u_{2} ) + g_{2} g_{6} g_{7} + g_{3} g_{4} ( g_{8} +\pi ) + g_{3} g_{7} ( g_{5} - u_{1} ) \bigr\} \bigr]. \end{aligned}$$

## Appendix 4

$$\begin{aligned} \frac{dU}{dt} = {}&\bigl( S - S^{1} \bigr) \biggl( \frac{\Lambda }{{S}} - \kappa p {I} {e}^{-\mu \tau } -\kappa p\omega {C} {e}^{-\mu \tau } -\mu + \frac{\eta {R}}{ {S}} \biggr) + \theta \kappa p {IS} {e}^{-\mu \tau } + \theta \kappa p\omega {CS} {e}^{-\mu \tau } \\ &{}- u_{1} {C}+ \kappa p {IS} \theta _{1} {e}^{-\mu \tau } + \kappa p\omega {CS} \theta _{1} {e}^{-\mu \tau } +\pi {C}- u_{2} {I}+ \beta C + \psi {I}- ( \mu + \eta ) {R}, \\ \frac{dU}{dt} ={}& \bigl( S - S^{1} \bigr) \biggl( \frac{\Lambda }{{S}} - \kappa p {I} {e}^{-\mu \tau } -\kappa p\omega {C} {e}^{-\mu \tau } - \frac{\Lambda }{{S}^{1}} + \kappa p {I} {e}^{-\mu \tau } +\kappa p\omega {C} {e}^{-\mu \tau } - \frac{\eta {R}}{ {S}^{1}} + \frac{\eta {R}}{ {S}} \biggr) \\ &{}-\mu {C}+\kappa p\omega {CS} {e}^{-\mu \tau } -\mu {I}+ \kappa p {IS} {e}^{-\mu \tau } -\mu {R}- \eta {R}- \sigma I, \\ \frac{dU}{dt} ={}& \bigl( S - S^{1} \bigr) \biggl[ \biggl( \frac{\Lambda }{{S}} - \frac{\Lambda }{{S}^{1}} \biggr) + \frac{\eta {R}}{ {S}} - \frac{\eta {R}}{ {S}^{1}} \biggr] -\mu {C} \biggl( 1- \frac{\kappa p\omega {S} {e}^{-\mu \tau }}{\mu } \biggr)\\ &{} - \mu I \biggl( 1- \frac{\kappa p {S} {e}^{-\mu \tau }}{\mu } \biggr) -\mu {R}-\eta {R}- \sigma I, \\ \frac{dU}{dt} ={}& \bigl( S - S^{1} \bigr) \bigl( S^{1} - S \bigr) \biggl[ \frac{\Lambda }{{S} {S}^{1}} + \frac{\eta {R}}{ {S} {S}^{1}} \biggr] -\mu {C} \biggl( 1- \frac{\kappa p\omega {S} {e}^{-\mu \tau }}{\mu } \biggr) \\ &{}- \mu I \biggl( 1- \frac{\kappa p {S} {e}^{-\mu \tau }}{\mu } \biggr) -\mu {R}-\eta {R}- \sigma I, \\ \frac{dU}{dt} ={}&{ - }\bigl( S - S^{1} \bigr)^{2} \frac{\Lambda }{{S} {S}^{1}} - \bigl( S - S^{1} \bigr)^{2} \frac{\eta {R}}{ {S} {S}^{1}} -\mu {C} \biggl( 1- \frac{\kappa p\omega {S} {e}^{-\mu \tau }}{\mu } \biggr)\\ &{} - \mu I \biggl( 1- \frac{\kappa p {S} {e}^{-\mu \tau }}{\mu } \biggr) -\mu {R}-\eta {R}- \sigma I. \end{aligned}$$

## Appendix 5

$$\begin{aligned} \frac{dV}{dt} = {}&\biggl( \frac{S - S^{*}}{S} \biggr) \frac{dS}{dt} + \biggl( \frac{C - C^{*}}{C} \biggr) \frac{dC}{dt} + \biggl( \frac{I - I^{*}}{I} \biggr) \frac{dI}{dt} + \biggl( \frac{R - R^{*}}{R} \biggr) \frac{dR}{dt}, \\ \frac{dV}{dt} = {}&\biggl( \frac{S - S^{*}}{S} \biggr) \bigl( \Lambda - \kappa p {IS} {e}^{-\mu \tau } -\kappa p\omega {CS} {e}^{-\mu \tau } -\mu {S}+\eta {R} \bigr)\\ &{} + \biggl( \frac{C - C^{*}}{C} \biggr) \bigl( \theta \kappa p {IS} {e}^{-\mu \tau } +\theta \kappa p\omega {CS} {e}^{-\mu \tau } - u_{1} {C} \bigr) \\ &{}+ \biggl( \frac{I - I^{*}}{I} \biggr) \bigl( \kappa p {IS} \theta _{1} {e}^{-\mu \tau } + \kappa p\omega {CS} \theta _{1} {e}^{-\mu \tau } +\pi {C}- u_{2} {I} \bigr)\\ &{} + \biggl( \frac{R - R^{*}}{R} \biggr) \bigl( \beta C + \psi {I}- ( \mu +\eta ) {R} \bigr), \\ \frac{dV}{dt} = {}&\bigl( S - S^{*} \bigr) \biggl( \frac{\Lambda }{S} - \kappa p {I} {e}^{-\mu \tau } -\kappa p\omega {C} {e}^{-\mu \tau } -\mu + \frac{\eta {R}}{ {S}} \biggr) \\ &{}+ \bigl( C - C^{*} \bigr) \biggl( \frac{\theta \kappa p {IS} {e}^{-\mu \tau }}{C} +\theta \kappa p\omega {S} {e}^{-\mu \tau } - u_{1} \biggr) \\ &{}+ \bigl( I - I^{*} \bigr) \biggl( \kappa p {S} \theta _{1} {e}^{-\mu \tau } + \frac{\kappa p\omega {CS} \theta _{1} {e}^{-\mu \tau }}{{I}} + \frac{\pi {C}}{ {I}} - u_{2} \biggr) \\ &{}+ \bigl( R - R^{*} \bigr) \biggl( \frac{\beta C}{R} + \frac{\psi {I}}{R} - ( \mu +\eta ) \biggr), \\ \frac{dV}{dt} = {}&\bigl( S - S^{*} \bigr) \biggl( \frac{\Lambda }{S} - \kappa p {I} {e}^{-\mu \tau } -\kappa p\omega {C} {e}^{-\mu \tau } - \frac{\Lambda }{{S}^{*}} + \kappa p {I} {e}^{-\mu \tau } +\kappa p\omega {C} {e}^{-\mu \tau } - \frac{\eta {R}}{ {S}^{*}} + \frac{\eta {R}}{ {S}} \biggr) \\ &{}+ \bigl( C - C^{*} \bigr) \biggl( \frac{\theta \kappa p {IS} {e}^{-\mu \tau }}{C} +\theta \kappa p\omega {S} {e}^{-\mu \tau } - \frac{\theta \kappa p {IS} {e}^{-\mu \tau }}{C^{*}} -\theta \kappa p\omega {S} {e}^{-\mu \tau } \biggr)\\ &{} + \bigl( I - I^{*} \bigr) \biggl( \kappa p {S} ( 1-\theta ) {e}^{-\mu \tau } + \frac{\kappa p\omega {CS} ( 1-\theta ) {e}^{-\mu \tau }}{{I}} + \frac{\pi {C}}{ {I}} - \kappa p {S} ( 1-\theta ) -\mu \tau \\ &{}- \frac{\kappa p\omega {CS} ( 1-\theta ) {e}^{-\mu \tau }}{{I}^{*}} - \frac{\pi {C}}{ {I}^{*}} \biggr)\\ &{} + \bigl( R - R^{*} \bigr) \biggl( \frac{\beta C}{R} + \frac{\psi {I}}{R} - \frac{\beta C}{R^{*}} - \frac{\psi {I}}{R^{*}} \biggr), \\ \frac{dV}{dt} ={}& \bigl( S - S^{*} \bigr) \biggl( \frac{\Lambda }{S} - \frac{\Lambda }{{S}^{*}} + \frac{\eta {R}}{ {S}} - \frac{\eta {R}}{ {S}^{*}} \biggr) + \bigl( C - C^{*} \bigr) \biggl( \frac{\theta \kappa p {IS} {e}^{-\mu \tau }}{C} - \frac{\theta \kappa p {IS} {e}^{-\mu \tau }}{C^{*}} \biggr) \\ &{}+ \bigl( I - I^{*} \bigr) \biggl( \frac{\kappa p\omega {CS} ( 1-\theta ) {e}^{-\mu \tau }}{{I}} - \frac{\kappa p\omega {CS} ( 1-\theta ) {e}^{-\mu \tau }}{{I}^{*}} + \frac{\pi {C}}{ {I}} - \frac{\pi {C}}{ {I}^{*}} \biggr) \\ &{}+ \bigl( R - R^{*} \bigr) \biggl( \frac{\beta C}{R} - \frac{\beta C}{R^{*}} + \frac{\psi {I}}{R} - \frac{\psi {I}}{R^{*}} \biggr), \\ \frac{dV}{dt} ={}& \bigl( S - S^{*} \bigr) \biggl[ \bigl( S^{*} - S \bigr) \frac{\Lambda }{SS^{*}} + \bigl( S^{*} - S \bigr) \frac{\eta {R}}{SS^{*}} \biggr] \\ &{}+ \bigl( C - C^{*} \bigr) \biggl[ \bigl( C - C^{*} \bigr) \frac{\theta \kappa p {IS} {e}^{-\mu \tau }}{CC^{*}} \biggr]\\ &{} + \bigl( I - I^{*} \bigr) \biggl[ \bigl( I^{*} - I \bigr) \frac{\kappa p\omega {CS} ( 1-\theta ) {e}^{-\mu \tau }}{II^{*}} + \bigl( I^{*} - I \bigr) \frac{\pi {C}}{II^{*}} \biggr] \\ &{}+ \bigl( R - R^{*} \bigr) \biggl[ \bigl( R^{*} - R \bigr) \frac{\beta C}{RR^{*}} + \bigl( R^{*} - R \bigr) \frac{\psi {I}}{RR^{*}} \biggr], \\ \frac{dV}{dt} ={}&{- }\bigl( S - S^{*} \bigr)^{2} \frac{\Lambda }{SS^{*}} - \bigl( S - S^{*} \bigr)^{2} \frac{\eta {R}}{SS^{*}} - \bigl( C - C^{*} \bigr)^{2} \frac{\theta \kappa p {IS} {e}^{-\mu \tau }}{CC^{*}}\\ &{} - \bigl( I - I^{*} \bigr)^{2} \frac{\kappa p\omega {CS} \theta _{1} {e}^{-\mu \tau }}{II^{*}} - \bigl( I - I^{*} \bigr)^{2} \frac{\pi {C}}{II^{*}} - \bigl( R - R^{*} \bigr)^{2} \frac{\beta C}{RR^{*}}\\ &{} - \bigl( R - R^{*} \bigr)^{2} \frac{\psi {I}}{RR^{*}}. \end{aligned}$$
